# Implementation of the Living Well During Pregnancy Telecoaching Program for Women at High Risk of Excessive Gestational Weight Gain: Protocol for an Effectiveness-Implementation Hybrid Study

**DOI:** 10.2196/27196

**Published:** 2021-03-18

**Authors:** Susan de Jersey, Nina Meloncelli, Taylor Guthrie, Hilary Powlesland, Leonie Callaway, Angela T Chang, Shelley Wilkinson, Tracy Comans, Elizabeth Eakin

**Affiliations:** 1 Centre for Clinical Research and Perinatal Research Centre Faculty of Medicine The University of Queensland Brisbane Australia; 2 Department of Nutrition and Dietetics Royal Brisbane and Women's Hospital Metro North Hospital and Health Service Brisbane Australia; 3 Allied Health Metro North Hospital and Health Service Brisbane Australia; 4 Department of Obstetric Medicine Royal Brisbane and Women's Hospital Brisbane Australia; 5 Faculty of Medicine University of Queensland Brisbane Australia; 6 Centre for Allied Health Research Royal Brisbane and Women's Hospital Metro North Hospital and Health Service Brisbane Australia; 7 School of Human Movements and Nutrition Sciences The University of Queensland Brisbane Australia; 8 Centre for Health Services Research Faculty of Medicine The University of Queensland Brisbane Australia; 9 School of Public Health Faculty of Medicine The University of Queensland Brisbane Australia

**Keywords:** implementation study, pregnancy, weight, nutrition, lifestyle intervention, physical activity

## Abstract

**Background:**

Despite comprehensive guidelines for healthy gestational weight gain (GWG) and evidence for the efficacy of dietary counseling coupled with weight monitoring on reducing excessive GWG, reporting on the effectiveness of interventions translated into routine antenatal care is limited.

**Objective:**

This study aims to implement and evaluate the Living Well during Pregnancy (LWdP) program in a large Australian antenatal care setting. Specifically, the LWdP program will be incorporated into usual care and delivered to a population of pregnant women at risk of excessive GWG through a dietitian-delivered telephone coaching service.

**Methods:**

Metrics from the RE-AIM (Reach, Effectiveness, Adoption, Implementation, and Maintenance) framework will guide the evaluation in this hybrid effectiveness-implementation study. All women aged ≥16 years without pre-exiting diabetes with a prepregnancy BMI >25 kg/m^2^ and gaining weight above recommendations at <20 weeks’ gestation who are referred for dietetic care during the 12-month study period will be eligible for participation. The setting is a metropolitan hospital at which approximately 6% of the national births in Australia take place each year. Eligible participants will receive up to 10 telecoaching calls during their pregnancy. Primary outcomes will be service level indicators of reach, adoption, and implementation that will be compared with a retrospective control group, and secondary effectiveness outcomes will be participant-reported anthropometric and behavioral outcomes; all outcomes will be assessed pre- and postprogram completion. Additional secondary outcomes relate to the costs associated with program implementation and pregnancy outcomes gathered through routine clinical service data.

**Results:**

Data collection of all variables was completed in December 2020, with results expected to be published by the end of 2021.

**Conclusions:**

This study will evaluate the implementation of an evidence-based intervention into routine health service delivery and will provide the practice-based evidence needed to inform decisions about its incorporation into routine antenatal care.

**International Registered Report Identifier (IRRID):**

DERR1-10.2196/27196

## Introduction

### Background

Excessive gestational weight gain (GWG) is a problem within the Australian obstetric population, occurring in 40% to 60% of pregnancies, particularly in the 50% of women already above a healthy weight prior to pregnancy [1]. This weight gain is associated with costly adverse medical and obstetric complications and contributes to the development of obesity in mothers and their offspring [2].

In Australia, comprehensive guidelines for healthy GWG from the Institute of Medicine (IOM [2]) have been widely promoted. Dietary counseling and weight monitoring have been shown to be effective in reducing GWG in research contexts and in routine antenatal care [3-5]. However, women already overweight before pregnancy are at greatest risk of excessive GWG [6], experience greater barriers to achieving a healthy weight gain, have lower confidence to achieve health goals [7], and have a need for more intensive support [8].

In addition, poor uptake of the typical clinical (face-to-face) model of dietetic counseling is seen in both research trials [9,10] and routine antenatal care services [11,12]. A face-to-face model of care to reduce GWG in overweight and obese pregnant women at our metropolitan tertiary hospital was poorly attended, and less than 10% of eligible women were referred [13,14]. Barriers to referral and engagement are likely multifactorial, with many women citing work commitments, timing, location [15], transport, and travel [16] as reasons for nonattendance.

Telephone counseling has been demonstrated as an effective and cost-effective solution for delivering positive weight management outcomes in a wide range of adult populations [17-19]. Individualized advice can be provided without requiring additional hospital visits and aligns with the preferences of women identified in formative work [20]. While various telephone-based interventions (either telephone counseling or text messages) have been trialed in the at-risk pregnant population, results have been varied, with limited reporting on the feasibility and cost-effectiveness of the approach within routine antenatal care settings [19,21]. This study aims to implement and evaluate the Living Well during Pregnancy (LWdP) program targeting pregnant women at risk of excessive GWG and delivered via telephone within the dietetic service in a large Australian tertiary setting. This program will be evaluated using metrics from the RE-AIM (Reach, Effectiveness, Adoption, Implementation, and Maintenance) framework, which includes effectiveness and implementation outcomes [22].

## Methods

### Study Design

The LWdP program will use a type 2 effectiveness-implementation hybrid design [23] to compare prospectively gathered data on participants enrolled in the LWdP program with a retrospective comparison group who was referred to the dietitian for pregnancy weight management. The setting is a tertiary metropolitan hospital that delivers approximately 4500 babies per year and offers 8 different models of antenatal care for both high- and low-risk pregnancies, including general practitioner shared care. These models of antenatal care will be consistent across the retrospective (preimplementation) and prospective (postimplementation) study periods. The retrospective control group will comprise pregnant women referred for dietetic care in the 12 months prior to the study period who meet the inclusion criteria. The prospective group will be consenting participants in the LWdP program referred for dietetic care during the 12-month study period. The RE-AIM evaluation framework will be used to understand the following about the LWdP program: reach and adoption (the number and representativeness of service referrers and participants), implementation (number of referrals, consent rates, fidelity of delivery, program completion rates, and costs of delivery), and effectiveness (maternal and neonatal outcomes) [22]. Evaluating the maintenance domain will not be possible within the time frame of the study. Ethics approval was granted from the Royal Brisbane and Women’s Hospital Human Research Ethics Committee (HREC/17/QRBW/159).

### Participants and Referral Pathways

All women aged ≥16 years without pre-exiting diabetes with a prepregnancy BMI >25 kg/m^2^ who are referred for dietetic weight management care during the study period will be eligible for participation. For the purpose of this evaluation, participants must be able to speak and read English sufficiently to allow program participation. Those women who do not meet these criteria will be provided with appropriate face-to-face dietetic care and not participate in this evaluation. Women will be referred into the program by their treating health care provider if they meet these criteria. Alternatively, women meeting these criteria will be able to self-refer into the program through a dedicated program website. Women who are engaged in the program who develop gestational diabetes will be provided the option to remain in the program or withdraw to the hospital’s gestational diabetes model of care.

### Engagement Strategy

Staff will be engaged in the planning phase of LWdP program implementation to identify preferred referral processes for each of the hospital’s 8 different antenatal models of care. This will inform the introduction of an outpatient dietetic referral form for the LWdP program alongside other dietetic services at the facility and the inclusion of the LWdP program referral in paperwork completed during women’s initial booking-in appointment. A program website will be developed to facilitate an online referral pathway, allowing both self-referral and referral by external antenatal health care providers located in the community of women under their care. A consumer engagement survey will be undertaken to inform the flexible program delivery hours, education needs, and topics of interest. Staff from each antenatal model of care will be provided with regular in-services, and the hospital’s media and communications team will be engaged to help build publicity for the program by featuring consumer stories in local newsletters, social media accounts, and local general practitioner communications.

### Screening and Consent

A waiver of participant consent was received as part of the ethics application to retrospectively access routinely collected clinical and administrative data from eligible women in the preimplementation group. For the postimplementation group, all women booked for an initial appointment in the LWdP program will be provided with an electronic link to the participant information sheet and electronic consent form with their participant workbook.

At the commencement of the program, the dietitian counsellor will discuss the evaluation process and answer any questions the participant may have. Program participants who consent to the evaluation will be requested to complete the consent form online in addition to having verbal consent recorded in their health record.

At the completion of the program, participants who verbally agreed to be involved in the evaluation but did not complete the consent form will be reminded to complete this online and a replacement link will be sent if required. Verbal consent will be documented on this second occasion. If no written consent form is received by the time of data analysis but two occasions of verbal consent are documented in the participant’s health record, consent to participate in the evaluation will be considered granted.

### LWdP Program

The LWdP program is aimed at supporting women at high risk of excessive weight gain during pregnancy to achieve GWG within recommendations [2] through the promotion of healthy eating and physical activity. Consistent with Australian dietary guideline recommendations for pregnancy and chronic disease prevention [24], participants will be encouraged to choose whole-grain cereal products, increase fruit and vegetable intake (5 servings of vegetables and 2 servings of fruits per day), choose foods with unsaturated fat, and limit discretionary food choices. They will also be encouraged to focus on eating the right amount of food through portion control and intuitive eating and choosing the right types of foods (minimally processed, low energy density, and high nutrient density). Over the course of the intervention, participants with no contraindications will also be encouraged to gradually undertake pregnancy-appropriate planned physical activity (such as walking, swimming, yoga) to accumulate 150 minutes per week [25]. An additional focus will be on reducing sitting time to contribute to energy expenditure. The goal is for women to track within the target healthy GWG range recommended by the IOM guidelines [2] based on their prepregnancy BMI through changing eating and activity behaviors.

Women referred to the program will be eligible for up to 10 telephone coaching calls over the course of their pregnancy that will usually span a period of 6 months (1 call each week for 1 month, then 1 call every 2 weeks for the next month, then 1 call per month for the next 4 months) and will also receive a participant workbook (Textbox 1). The initial telephone call will be approximately 60 minutes in duration, with subsequent calls lasting approximately 30 minutes, with an emphasis on motivational interviewing [26] and tailored health behavior coaching [27]. The program has been adapted for pregnancy from the Healthy Living after Cancer program [28] and informed by previous formative work with women participating in the New Beginnings Healthy Mothers and Babies study at the same institution [1,20,29,30]. Specifically, the program will target behavioral constructs and consumer experiences gained from prior research with the study population [1,20,29,30]. The intervention is grounded in the social cognitive theory constructs of self-efficacy, social support, and outcome expectancies [31] and emphasizes developing skills using behavior change strategies—goal setting, self-monitoring, identifying potential barriers and problem solving, identifying social support, stimulus control, mindful eating, positive self-talk, and self-reward. The protocol for each call includes an assessment of progress, problem solving, advice/education, collaborative goal setting/goal progression, and development/revision of a behavior-specific action plan. The program topics have been mapped to the refined taxonomy of behavior change techniques [32], as shown in Multimedia Appendix 1.

**Living Well during Pregnancy participant workbook content.**

Introduction to the Living Well during Pregnancy program

- Coach introduction, scheduling, and discussion of the importance of healthy weight gain in pregnancy

Section 1: Planning for success

- Participant aims
- SMART (Specific, Measurable, Achievable, Relevant, and Timed) goal setting and action plan
- Problem solving
- Tracking tools for eating behaviors, physical activity, weight gain, and meal planning

Section 2: What to eat and how much

- Benefits of healthy eating and recommendations in pregnancy
- Important nutrients (including supplementation)
- Portion guide
- Self-monitoring

Section 3: Fat, fiber, and food safety

- Increasing intake of fruits, vegetables, and whole grains
- Reducing saturated fat intake
- Food safety

Section 4: Keeping active

- Safe exercise during pregnancy
- Planning activity
- Pelvic floor exercises
- Reducing sitting time

Section 5: Healthy pregnancy weight gain and energy balance

- Energy density and food substitutions
- Strategies to slow rapid weight gain
- Healthy snacking

Section 6: Meal planning and preparation

- Creating healthy meals
- Grocery shopping planning
- Plating portion guide
- Strategies for healthy eating away from home

Section 7: Mindful eating

- Breaking the dieting cycle
- Hunger cues
- Mindful eating
- Overeating triggers
- Food and emotions

Section 8: Celebrating success and planning ahead

- Planning ahead
- Potential obstacles
- Celebrating success
- Getting support from others
- Healthy eating during breastfeeding

Accredited Practising Dietitians with experience in providing antenatal care services who have undergone additional training in motivational interviewing will be trained in the delivery of the program. A training manual detailing the program protocol including call transcripts will be provided to all dietitians delivering the program. This additional training includes program philosophy, person-centered care, work shadowing, and the delivery of each call with provision of feedback from the trainer and a checklist of training requirements prior to commencing program delivery. At the completion of each call, the dietitian coach will be required to complete a fidelity checklist to assess the degree to which the call aligned with the protocol.

The program is structured to offer flexibility in the scheduling of call times outside of usual business hours, with early morning and evening times available. Additionally, a caseload continuity of care model will be offered where each participant is allocated one dietitian coach for their pregnancy. Upon booking the first coaching session, each participant will be provided with a welcome letter with information about her coach to assist with establishing rapport.

### Intervention Procedures

The participant workbook will be used to structure the order of intervention topics for each telecoaching session (Textbox 1). Participants will be encouraged to commence with a focus on healthy eating, as there is strong evidence to support dietary interventions for moderating pregnancy weight gain [33,34]. However, the intervention is tailored to meet individual participant’s needs and motivations.

In the telephone-delivered program, the calls are structured into three phases (Multimedia Appendix 2), with weekly, biweekly, and then monthly phone calls. Frequent contact at the beginning of the program provides more intensive support for behavior change from the outset. Less frequent contact in the latter part of the program facilitates participants’ autonomy and confidence in consolidating and maintaining lifestyle changes such that they will (optimally) become lifelong habits.

### Usual Care

The retrospective group was provided with traditional, unstructured face-to-face dietetic care. Women were offered individual appointments with the dietitian in a dedicated clinic within the maternity outpatient department. An initial appointment was booked for 40 minutes, with review appointments allocated 20 minutes. There was no guarantee of the same dietitian at each review appointment, and review appointments were booked based on clinical judgment in negotiation with the woman. No specified educational content or strategies were provided.

### Data Collection

Data will be collected by study-trained dietitian research assistants. Research Electronic Data Capture (REDCap), a secure online survey and data capture tool, will be used to record details of each telephone counselling session including progress, clinical information, and fidelity to the call procedures and topics. Routinely collected maternal and infant data will be accessed from medical records.

#### Primary and Secondary Outcomes

Outcomes are shown in Table 1 along with corresponding RE-AIM indicators and assessment tools. Details of validated tools to be used are provided below. Primary outcomes will include referrals and referral sources, uptake among eligible patients, withdrawal rates and reasons (where available), completion rates, average number of calls per participant, average length of calls per participant, completion of pre-post program assessments, staff and participant satisfaction, and program delivery costs. Secondary outcomes will include routinely collected delivery and pregnancy outcomes as well as patient-reported outcomes collected via online questionnaires at the commencement and completion of the program (anthropometrics, and behavioral and psychosocial factors). If a participant does not complete these assessments online, a follow-up telephone call will be completed by study staff to gather information via the telephone.

**Table 1:** Primary and secondary outcomes, assessment tools, and RE-AIM (Reach, Effectiveness, Adoption, Implementation, and Maintenance) framework indicators.

RE-AIM indicator and description of outcome					Collection method/assessment tools
**Reach and representativeness (primary outcome)**					
	Percentage of eligible women referred			Administrative data	
	Percentage of referred women taking up the service			Administrative data	
	Number of sessions attended			LWdP^a^ program REDCap^b^ database	
	Participant characteristics			LWdP program REDCap database	
**Implementation (primary outcome)**					
	Completion of pre- and postprogram assessments			Questionnaires	
	Staff survey			Questionnaires	
	Completion rates			LWdP program REDCap database	
	Withdrawal rates			Medical records and LWdP program REDCap database	
	Reasons for withdrawal			Questionnaire	
	Coaching call fidelity			Self-assessment database	
	Interventionist characteristics and allocated participants			Staff administrative database	
	Implementation strategies			Mapped to Expert Recommendations for Implementing Change (ERIC) strategies [35]	
**Adoption and maintenance (primary outcome)**					
	Referral source and number of referrals			Medical records and administrative data	
	Staff survey			Questionnaire	
	Adherence to implementation protocol			LWdP program REDCap database	
**Effectiveness (secondary outcome)**					
	Anthropometric outcomes				
		Weight, height, BMI	Medical records and self-reports		
		Gestational weight gain	Medical records, classified according to Institute of Medicine recommendations [2] (adjusted to 36 weeks)		
	Behavioral outcomes				
		Dietary intake	Fat and fiber behavior questionnaire [36]		
		Physical activity	Active Australia Survey [37]		
		Sedentary behavior	International Physical Activity Questionnaire [38]		
	Psychosocial outcomes			Intuitive eating scale [39]	
	Delivery and pregnancy outcomes				
		Gestational diabetes mellitus (diagnosed according to the Queensland Health Clinical Guideline for Gestational diabetes mellitus [40]); pregnancy-induced hypertension (excluding pre-eclampsia and HELLP); pre-eclampsia; mode of delivery; delivery complications; birth weight: macrosomia (>4000 g) and large for gestational age (>90th percentile based on Australian population [41]); neonatal morbidity, treatment, and admission to special or intensive care	Medical records		
	Participant satisfaction			Semiquantitative survey	
	Staff satisfaction			Semiquantitative survey	
	Cost			Hospital financial and outcome records	

^a^LWdP: Living Well during Pregnancy.

^b^REDCap: Research Electronic Data Capture.

#### Withdrawal Rates and Reasons

Women who withdraw from the program after receiving at least one coaching call will be sent a text message with a link to a short electronic survey. This anonymous survey will evaluate satisfaction with the program and reasons for program withdrawal.

#### Staff Survey

The understanding and acceptability of the program by staff (including clinicians and administrative personnel) will be assessed via a survey 2 months prior to completion of the evaluation period. Both electronic and paper-based surveys will be available. Electronic links to the online survey will be disseminated to staff from line managers. Paper-based surveys will be available in staff lunchrooms with collection boxes available for return of the survey. Administrative staff will be asked about referral and booking processes, whereas clinicians will be asked about their referral practices, program understanding, and barriers to referral. Survey responses will be provided on a 5-point Likert scale.

#### Dietary Intake and Behavior

The fat and fiber behavior questionnaire (FFBQ) is a 20-item questionnaire used to assess dietary behaviors (frequency of consumption, use of food items, and food categories) that has been validated against a food frequency questionnaire in the adult Australian population and is able to detect changes in fiber and fat-related intake behaviors [36]. It provides information on general food patterns rather than specific energy and macronutrient intake. Nine items relate to the frequency of consumption of particular high-fat or high-fiber foods, measured on a 5-point Likert scale (ranging from 5=never to 1=6 or more days per week). Nine items ask about behaviors related to cooking, eating, or choosing foods, such as type of dairy products or bread, measured on a 5-point Likert scale (ranging from 1=never to 5=always) with a “not applicable or do not know” option for those who do not eat a particular food or are not aware of specific cooking methods [36]. Two items assess the number of servings of fruits (1 item) and vegetables (1 item) consumed each day [36]. These 2 items contribute to the fiber and total FFBQ scores and are also valid stand-alone measures of fruit and vegetable intake [42]. The FFBQ is easy to score and administer, provides a good alternative to food frequency questionnaires in interventions, and has been used previously with pregnant women [20].

The Intuitive Eating Scale-2 is a 23-item tool that measures individuals’ tendency to follow their physical hunger and satiety cues when determining when, what, and how much to eat [39]. Participants select the option that best describes their attitude or behavior to each item rated on a 5-point Likert scale (ranging from 1=strongly disagree to 5=strongly agree).

#### Physical Activity and Sedentary Behavior

The Active Australia Survey consists of 8 questions designed to assess the frequency and duration of walking and performing moderate and vigorous physical activity in the past week [37]. The total number of sessions and minutes of physical activity will be treated as continuous variables. Values greater than 840 minutes will be recoded to this value to avoid over-reporting in accordance with recommendations for use of survey items [23]. A single item from the International Physical Activity Questionnaire [38] will assess sedentary time. This item assesses the total duration of sitting time on a weekday in the past 7 days [38]. These questionnaires will all be administered electronically and verified by the dietitian.

#### GWG

Pregnancy weight data have been collected routinely at the study hospital since 2015. GWG will be calculated by subtracting self-reported prepregnancy weight from weight recorded at 36 weeks’ gestation [43]. Self-reported prepregnancy weight has been demonstrated to be an accurate depiction of weight at conception for most women [44]. For women who deliver prior to their 36-week appointment, the last recorded weight will be used to estimate GWG and will be adjusted for in multiple variable analysis. GWG will be categorized as inadequate, within range, or excessive according to prepregnancy BMI ranges recommended by the IOM [2] and adjusted for the 36-week measure.

### Analysis

#### Economic Evaluation

An economic analysis from a health services perspective will be conducted following the International Society for Pharmacoeconomics and Outcomes Research (ISPOR) guidelines for modeling [45]. The fixed costs of implementing the LWdP program (ie, expenditures required to deliver the program and train dietitians in health coaching) will be documented. Dollar values will be assigned to these resources using publicly available information such as appropriate salary rates for the time of personnel involved in the above activities and commercial costs for the production of any training materials and program booklets. Fixed costs will be allocated equally over all participants who consented to participate in the program. Variable costs (ie, those that are proportional to the volume of service provided) will be allocated in proportion to the stage of the program reached by individuals [46].

The costs of different birth outcomes will be determined from the Royal Brisbane and Women’s Hospital’s unit costing based on the previous 12 months of administrative data covering all births occurring in this time frame. Costs will be stratified by BMI category and gestational diabetes status. As cost data of this nature generally have asymmetric distributions and heteroskedastic errors [47], an appropriate statistical model (eg, generalized linear model, generalized linear mixed model, or generalized estimating equation) for comparing the costs of birth by gestational diabetes status will be determined once the underlying distribution and quality of the data are assessed. As quality of life is not measured within the trial or available in the preintervention administrative data, utility weights will be assigned to birth outcomes using relevant literature sources [48,49].

A cost-utility analysis will be undertaken with the primary analysis from the perspective of the health care system. This perspective is the most commonly used economic evaluation method in Australia for policy makers [50] and will allow comparisons across other sectors to identify the relative cost-effectiveness of the intervention for funding proposals. An incremental cost-effectiveness ratio (ICER) will be calculated using the following formula:

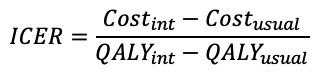



where *int* is the new intervention, *usual* represents usual care, and *QALY* stands for quality-adjusted life-year.

The model will include sensitivity analysis of key parameters, and outputs will include cost-effectiveness acceptability curves; these will display the probability of cost-effectiveness at varying thresholds of net monetary benefit.

#### Statistical Analysis

Primary (program implementation) outcomes (reach, uptake, and retention) will be reported descriptively and compared with the outcomes of a retrospective face-to-face comparison group. Where corresponding data are available for any secondary outcomes (such as GWG), results from the retrospective group will be compared with those of the prospective group. Chi-square and independent sample *t* tests will compare outcomes between retrospective and prospective groups. Analyses of secondary (program effectiveness) outcomes in the prospective group will be by mixed models, which will allow for repeated measures (baseline and follow-up) and include all participants with baseline data (including those with missing data at follow-up), with adjustment for predictors of dropouts to minimize selection bias. The relationship between intervention dose (number of calls received) and secondary behavioral and clinical outcomes will be examined. The optimal model will be selected using both indices of fit and model interpretability and parsimony. Outcomes will be reported as per protocol and intention-to-treat. Per protocol is defined as any participant who completed 4 or more telephone counseling sessions. Sensitivity of conclusions to missing data assumptions will be evaluated.

## Results

The LWdP program commenced in February 2018. The prestudy period for retrospective data inclusion was from October 2016 to October 2017 and includes data from 49 women. The poststudy period for data collection commenced when the program was initiated in February 2018 and the last included participant for data analysis was enrolled in August 2019. A total of 152 women consented to have their data included in the poststudy period. Data collection of all variables was finalized in December 2020. Data cleaning has commenced and data analysis will be completed by June 2021.

## Discussion

Gaining too much weight in pregnancy is an issue in Australia and occurs more frequently with women who begin their pregnancy above a healthy weight [1,6]. Although dietary counseling and weight monitoring are effective in reducing excessive GWG [34], intensive support is needed for those who experience significant barriers to achieving healthy weight gain [29]. While the evidence for pregnancy weight management interventions exists [34], translating evidence into usual antenatal care services is challenging. Furthermore, traditional face-to-face models of care are typically poorly attended and provide many women with an additional barrier to accessing support.

The LWdP telephone counseling program is aimed at supporting women at high risk of excessive weight gain during pregnancy to achieve GWG within the recommendations. Dietetic counseling will be used to promote healthy eating and physical activity, consistent with the Australian dietary guidelines, during 10 telephone coaching calls. This paper presents a pragmatic implementation and evaluation of the LWdP program to reduce excessive GWG using the RE-AIM evaluation framework to determine considerations for dissemination, scalability, and sustainability. This study will extend the current knowledge base that telephone counseling is cost-effective and results in desired behavior changes in many other adult populations [17,28], adding evidence regarding cost-effectiveness and feasibility in a population at risk of excessive GWG, as well as document clinical effectiveness when delivered through routine antenatal care. Furthermore, the incorporation of a full economic analysis will allow the relative costs and benefits of this program to be assessed. This information is important to funders and policy makers to understand the implications of scaling up the intervention.

A potential limitation of the study is that it will be delivered at a single (Queensland) institution. However, it is hoped that with 6% of the approximately 68,000 Queensland births taking place at this hospital each year and the hospital’s antenatal population being generally representative of the state’s demographics [1], the generalizability of the study findings will be increased. The evaluation metrics gathered through the RE-AIM framework will inform future adoption and dissemination considerations to other centers with a different resourcing context. Further, while the use of a retrospective control group and change-over-time design introduces limitations in data interpretation when compared with a randomized controlled trial, applying a pragmatic implementation approach provides a logical, “real-world” approach that is useful in health service research and implementation science. Additionally, the reliance on the self-reported prepregnancy weight and nutrition intake and physical activity measures, as well as self-reported fidelity adherence to the dietitian coaching calls, introduces the potential for recall bias and, hence, biased analyses. This is a common concern for GWG, nutrition, and physical activity studies, but given the purpose, size, and budget of this study, more detailed assessments were not considered feasible. Importantly, the study’s strengths include the applied nature of the implementation, robust health services data available, and integration into routine care. By evaluating the implementation of an evidence-based intervention into routine health service delivery will provide the practice-based evidence needed to inform decisions about its incorporation into routine antenatal care.

## Appendix

Multimedia Appendix 1 Living Well during Pregnancy intervention content mapped to the taxonomy of behavior change techniques (v1).

Multimedia Appendix 2 Intervention phases, call frequency, and call objectives of the Living Well during Pregnancy telephone counselling program.

## Abbreviations

ERIC: Expert Recommendations for Implementing Change
FFBQ: fat and fiber behavior questionnaire
GWG: gestational weight gain
ICER: incremental cost-effectiveness ratio
IOM: Institute of Medicine
ISPOR: International Society for Pharmacoeconomics and Outcomes Research
LWdP: Living Well during Pregnancy
RE-AIM: Reach, Effectiveness, Adoption, Implementation, and Maintenance
REDCap: Research Electronic Data Capture
