# Supplementary material for: Implementation of the Living Well During Pregnancy Telecoaching Program for Women at High Risk of Excessive Gestational Weight Gain: Protocol for an Effectiveness-Implementation Hybrid Study
Source: JMIR Res Protoc. 2021 Mar 18;10(3):e27196. doi: 10.2196/27196 (PMC8086782; doi:10.2196/27196)
Supplement: Multimedia Appendix 1 [file resprot_v10i3e27196_app1.docx]

Living Well during Pregnancy intervention content mapped to the taxonomy of Behaviour Change Techniques (v1) [32]

| Behaviour change technique (listed by taxonomy number) | Living Well during Pregnancy example components |
| --- | --- |
| 1.Goals and planning | |
| 1.1 Goal setting (behaviour) | Planning for success, SMART goal setting of eating behaviour, physical activity and sedentary behaviour |
| 1.2 Problem solving | Identifying what is getting in the way and coming up with solutions |
| 1.3 Goal setting (outcome) | Goal setting GWG targets in relation to pre-pregnancy BMI and GWG progress |
| 1.4 Action planning | Action planning in relation to SMART behavioural goals |
| 1.5 Review behaviour goals | Each telephone call, coach and participant review behavioural goal progress. |
| 1.6 Discrepancy between current behaviour and goal | Coach to build discrepancy between goals and behaviour *“Let’s think back to what you wanted to achieve from the Living Well during Pregnancy program. Is what you’re doing currently with your physical activity/nutritional intake getting you there? Are you happy with things the way they are?”* |
| 1.7 Review outcome goal | Each telephone call, coach and participant review GWG progress in relation to goal. |
| 2.0 Feedback and monitoring | |
| 2.2 Feedback on behaviour | Each call commenced with checking behavioural tracking, review and feedback on goals*“We’re going to check how you went with your healthy eating goal and tracking, discuss some of the content in the* ***Healthy Eating*** *section and work together to set a* ***healthy eating*** *goal.”* |
| 2.3 Self-monitoring of behaviour | Participant monitoring of dietary intake on healthy eating tracker and physical activity on activity tracker |
| 2.4 Self- monitoring of outcome of behaviour | Monitoring of GWG on personalised pregnancy weight gain chart |
| 2.7 Feedback on outcome of behaviour | Each call to involved checking GWG tracking and provide feedback on progress *“We’re going to see how you are going with you weight gain tracking and where this is in relation to your recommendations and goals”* |
| 3.0 Social support | |
| 3.1 Social support (unspecified) | Activating support for living well. Coach to discuss the steps participants can take to help activate best supports. For example, participants could ask their friends and family to provide a healthy food option at events or gatherings or to choose restaurants that have healthy eating options when they eat out together. Or, they could ask their friends and family to join them in activities such as bushwalking instead of more sedentary activities like seeing a movie together. Participants are encouraged to write this down in the space provided in the workbook. Participant to set goal for activating support. |
| 4.0 Shaping knowledge | |
| 4.1 Information about antecedents | Coach discusses with participants factors associated with achieving healthy eating, physical activity and GWG goals such as planning, problem solving, enlisting support, self-awareness. |
| 4.2 Re-attribution | Discuss triggers to unhelpful behaviour, potential causes, and mindfulness around cues |
| 5.0 Natural consequences | |
| 5.1 Information about health consequences | Discuss and provide written information about consequences of unhealthy eating, physical inactivity and unhealthy GWG |
| 7.0 Associations | |
| 7.1 Prompts/cues | Set alarm on phone for activity, put note on television remote to stand in add breaks |
| 8.0 Repetition and substitution | |
| 8.2 Behaviour substitution | Swapping unhealthy behaviours with alternative healthy options to reduce nutrient poor foods and sedentary behaviours |
| 8.3 Habit formation | Prompt participants to incorporate positive health behaviours such as meal planning, shopping and dietary balance using plate model within their routine permanently. |
| 8.4 Habit reversal | Prompt participants to replace unhelpful habits with alternative positive ones such as a short 10 minute walk after lunch or dinner, planning healthy mid-meals instead of relying on vending machines |
| 8.7 Graded tasks | Negotiating with participants small changes to achieve bigger goals, e.g. achieving 10 minute walk daily before increasing |
| 9.0 Comparison of outcomes | |
| 9.2 Pros and cons | Coach to support participants to with building the discrepancy between their current behaviours and their aims, identifying reasons for change and articulating reasons not to change. |
| 10 Reward and threat | |
| 10.4Social reward | Coach to provide positive reinforcement through verbal reward during each call for changes made and goal attainment. Assessment made through progress review at commencement of call. |
| 10.7 Self incentive | Planning for success within phone calls and workbook by including incentives for achieving behavioural goals. |
| - 1. Self-reward | Coach helping the participant to identify meaningful rewards to reinforce goals attained. |
| 12 Antecedents | |
| 12.1 Restructuring the physical environment | Coach supporting participant to change environmental cues to reduce availability of unhealthy foods, increase availability of healthy options and increasing opportunities for physical activity. E.g keeping healthy snack options in draw at work, removing biscuits eye height in pantry cupboard. |
| 12.2 Restructuring the social environment | Coach supporting participant to change social cues to facilitate goal attainment for healthy eating, and physical activity such as reducing the number of occasions eating out, increasing social activities that involve physical activity eg. Going for a walk with a friend instead of a movie |
| 12.3 Avoidance/reducing exposure to cues for the behaviour | Coach supporting participant to identify cues and barriers to achieving goals and discussion opportunity to avoid these such as grocery shopping after a meal rather than when hungry, mindless eating, eating while watching television |
| 12.4 Distraction | Coach supporting participant to identify distraction activities from food triggers, cravings and negative thoughts, including workbook activity. E.g Stop, breathe, think; environmental focus; bridging objections; mental games |
| 13. Identity | |
| 13.2 Framing/Reframing | Program philosophy of non-diet, sustainable changes to continue lifelong. Reframing thoughts about dieting and foods. |
| 15. Self-belief | |
| 15.1 Verbal persuasion | Coach to focus on positive attributes of participant and advice person on their ability to successfully change eating or activity behaviour. |
| 15.3 Focus on past success | Coach to guide participant to recall previous successful behaviour change situations, discuss context and factors associated with success. |
| 15.4 Self-talk | On weekly goal reflection, coach to prompt for positive experiences with goal attainment. Discuss and advise on positive self-talk before and during desire behaviour. |
