# Supplementary material for: Implementation of the Living Well During Pregnancy Telecoaching Program for Women at High Risk of Excessive Gestational Weight Gain: Protocol for an Effectiveness-Implementation Hybrid Study
Source: JMIR Res Protoc. 2021 Mar 18;10(3):e27196. doi: 10.2196/27196 (PMC8086782; doi:10.2196/27196)
Supplement: Multimedia Appendix 2 [file resprot_v10i3e27196_app2.docx]

Appendix 2. Intervention phases, call frequency and call objectives of the Living Well during Pregnancy telephone counselling program

| Phase | Call frequency | #Calls | Purpose | Objectives |
| --- | --- | --- | --- | --- |
| Phase one Month 1 | Weekly | 1-4 | Rapport-building, engagement, skill-building, education | - Build rapport - Program overview and participant aims - Feedback on pre-program assessment to build motivation to change - Build participant engagement and increased self-awareness of current health behaviours through self-monitoring - Understand importance of healthy eating, physical activity and healthy weight in pregnancy - Understand and begin using behaviour change skills, setting goals, tracking, problem- solving, identifying benefits, rewarding success |
| Phase two Month 2 | Fortnightly | 5-6 | Putting it into practice | - Progress goals - Add new target behaviours - Review progress, reinforce success, identify benefits and problem-solve barriers - Ongoing education - Build participant self-efficacy, knowledge and confidence through feedback and positive reinforcement |
| Phase 3 Months 3-6 | Monthly | 6-10 | Consolidation and maintenance | - Use of supports and strategies for maintaining changes - Move to participants taking the lead in progressing goals and tracking their behaviour - Feedback on post program assessment to reinforce participant’s progress and success - Closure |
